# Uterine Myxoid Leiomyosarcoma with Tumor Embolism Extending into the Right Atrium

**DOI:** 10.1155/2015/316262

**Published:** 2015-02-02

**Authors:** Hiromi Imai, Hiroshi Yagi, Kaoru Okugawa, Hironori Kenjo, Tatsuhiro Ohgami, Yoshiaki Kawano, Eisuke Kaneki, Akimasa Ichinoe, Kazuo Asanoma, Hideaki Yahata, Kenzo Sonoda, Hiroaki Kobayashi, Tsunehisa Kaku, Kiyoko Kato

**Affiliations:** ^1^Department of Obstetrics and Gynecology, Graduate School of Medical Sciences, Kyushu University, Fukuoka 812-0054, Japan; ^2^Department of Obstetrics and Gynecology, Faculty of Medicine, Kagoshima University, Kagoshima 890-8520, Japan; ^3^Department of Health Sciences, Graduate School of Medical Sciences, Kyushu University, Fukuoka 812-0054, Japan

## Abstract

Uterine myxoid leiomyosarcoma (MLMS) is an extremely rare variant of uterine leiomyosarcoma; only 56 cases were reported from 1982 to 2013. Uterine MLMS is characterized by a myxoid appearance and highly malignant behavior. We herein report a case involving a 65-year-old woman with uterine MLMS with a large tumor embolism that reached the right atrium. A total abdominal hysterectomy, bilateral salpingooophorectomy, and tumor embolism resection with the use of a heart-lung machine were performed. Epirubicin-ifosfamide chemotherapy in the adjuvant setting led to reductions in both the tumor emboli and peritoneal dissemination. The patient retained a good quality of life for 10 months after the initial surgery. She then developed progressive disease despite treatment with pazopanib. She died of her disease 14 months after the initial surgery. Although complete surgical resection of the tumor is desirable, tumor reduction surgery followed by adjuvant chemotherapy might help to retain a good quality of life. This is the first reported case of a primary uterine MLMS with tumor emboli.

## 1. Introduction

Myxoid leiomyosarcoma (MLMS) is a rare uterine tumor. Uterine MLMS was first described in 1982 by King et al. [1], and only 56 cases were reported in the literature from 1982 to 2013 [1–9]. MLMS is characterized grossly by a gelatinous cut surface and circumscribed border, microscopically by a large amount of myxomatous stroma and a low mitotic count, and clinically by highly malignant behavior despite a low mitotic index [1]. However, the diagnosis of uterine MLMS is now based on the gross and microscopic features of the tumor rather than the mitotic index because some cases have been shown to have a high mitotic index [2–9]. We herein report a case of an aggressive uterine MLMS with a large tumor embolism that reached the right atrium.

## 2. Case Presentation

**Figure 1:**
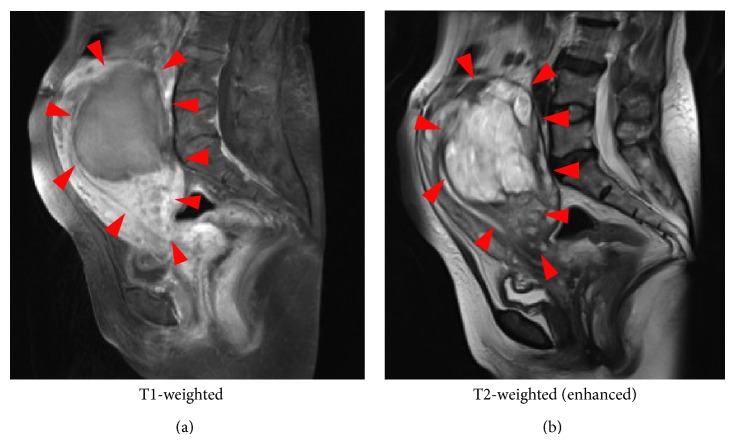
Frontal pelvic magnetic resonance imaging demonstrated a lobular tumor with low T1- and high T2-signal intensity on the posterior wall of the uterus.

**Figure 2:**
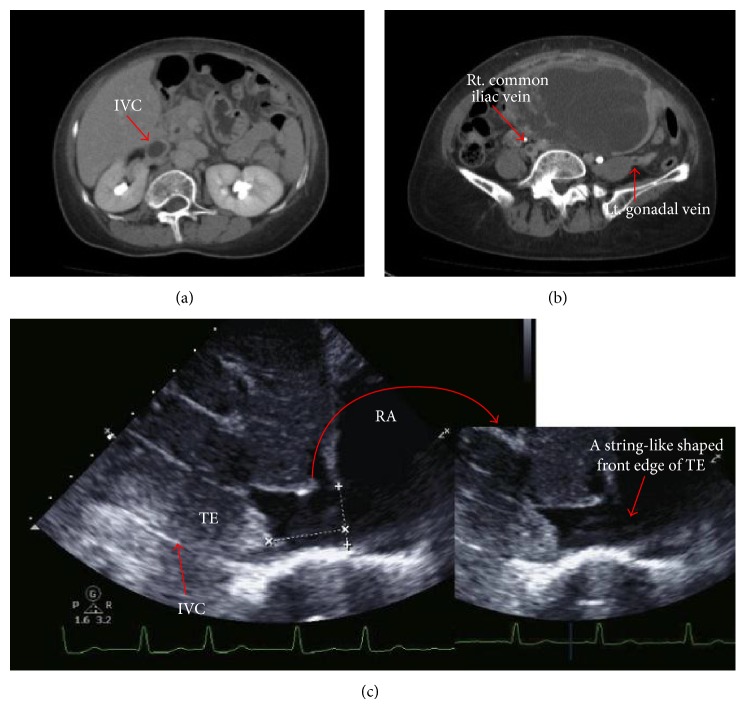
(a, b) Axial computed tomography revealed tumor emboli extending from the uterus to the inferior vena cava. (b) Right common iliac and left gonadal veins. (c) Cardiac ultrasonography revealed a string-like front edge of the tumor embolism inside the right atrium. IVC, inferior vena cava; TE, tumor embolism; RA, right atrium.

A 65-year-old woman (gravida 5, para 3) presented with lower abdominal pain. Computed tomography revealed a pelvic tumor, and she was referred to us for further examination. At the first visit, the patient's general condition was good, her vital signs were stable, and her body mass index was 21.8 kg/m^2^. The patient's medical and family histories were unremarkable. On pelvic examination, a firm, nontender, approximately 15 cm diameter mass was palpated. Magnetic resonance imaging demonstrated a lobular tumor with low T1- and high T2-signal intensity on the posterior wall of the uterus (Figure 1). Transabdominal ultrasonography and computed tomography revealed a 16 cm diameter uterine tumor with linear and nodular calcifications. After intravenous contrast administration, the lesion exhibited peripheral enhancement with centripetal filling. Additionally, a tumor embolism that extended from the uterus to the right external, internal, and common iliac veins and inferior vena cava (IVC) and reached the right atrium (RA) was detected (Figures 2(a) and 2(b)). Cardiac ultrasonography showed that the front edge of the tumor embolism inside the RA was string-like in appearance (Figure 2(c)). Smear cytology of the endometrium was negative. The serum lactate dehydrogenase level was 328 U/mL (reference range, 119–229 U/mL), and all other laboratory data, including the CA125, CA19-9, and carcinoembryonic antigen levels, were within normal limits. Based on these findings, we suspected a malignant uterine tumor, such as a leiomyosarcoma, with a massive tumor embolism. An urgent operation was planned because of the risk of a sudden cardiac event.

A one-stage operation was performed by a combined team of gynecologists and general and cardiac surgeons. Surgical exposure was accomplished through a sternotomy and median laparotomy. Examination of the abdominal cavity revealed a 16 cm soft uterine mass as well as a soft intraligamentary tumor in the bilateral uterus. First, hysterectomy and bilateral salpingooophorectomy were performed. Next, the IVC was exposed by complete mobilization of the right hemiliver, and the falciform and coronary ligaments were transected. After cannulation of the aorta, superior vena cava, and femoral vein, cardiopulmonary bypass and cardioplegia were established. After clamping the superior vena cava and IVC at the entrance of the RA and pulmonary artery, respectively, the RA was opened. The string-like front edge of the tumor embolism inside the RA was resected. The IVC was clamped above the gonadal artery and opened. The tumor embolism, which measured 7 cm in length and 2 cm in width, was then resected. Next, the IVC and RA were reconstructed and cardiopulmonary bypass was uneventfully weaned. A tumor embolism in the right common iliac vein and bilateral gonadal artery and vein was also resected. Residual disease was present in the pelvic venous plexus upon completion of the surgery.

**Figure 3:**
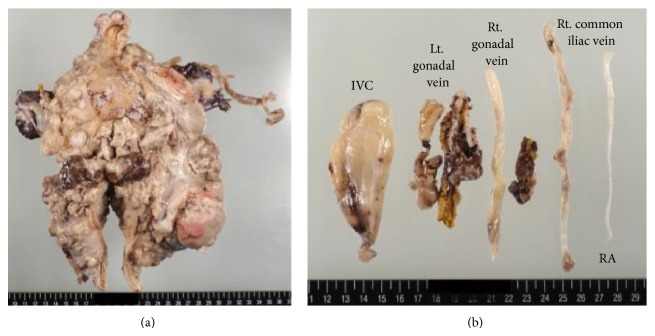
Operative specimen. (a) The 17 cm uterine tumor was soft with a gelatinous cut surface. (b) Intravascular components of the tumor. IVC, inferior vena cava.

**Figure 4:**
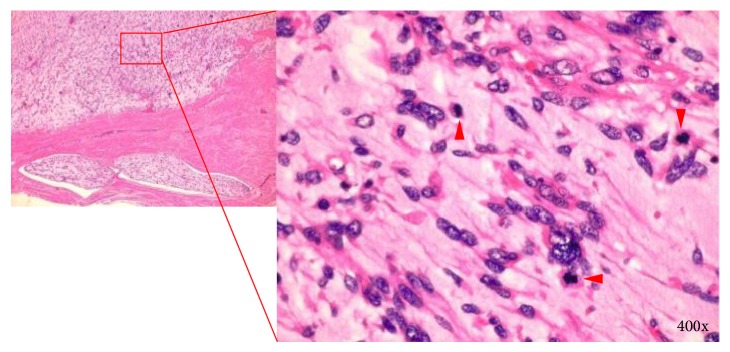
Infiltrating border of the tumor. Arrowheads indicate mitotic figures.

Macroscopically, the 16 cm uterine tumor was soft with a gelatinous cut surface (Figure 3(a)). The tumor emboli were also soft (Figure 3(b)). Microscopically, the tumor sections showed a proliferation of spindle- to polygonal-shaped malignant cells with hyperchromatic and pleomorphic nuclei; the cells were accompanied by abundant chondromyxoid stroma and slit-like vessels (Figure 4). Massive coagulative necrosis was also seen. The mitotic count was relatively high (20 mitotic figures per 10 high-power fields). Lymphovascular permeation was frequently observed. Bulbous and rounded infiltration into the whole thickness of the myometrium was observed throughout almost the entire uterine wall (Figure 4). Immunohistochemically, the malignant cells were focally positive for alpha-smooth muscle actin, HHF35, and CD10, but negative for desmin, estrogen receptor, and progesterone receptor. The MIB-1 labeling index was approximately 40%. These histological findings led to the diagnosis of uterine MLMS with tumor emboli and metastasis to the bilateral adnexa.

The patient was managed in the intensive care unit immediately postoperatively and moved to the general ward on postoperative day 2. Twenty-two days postoperatively, computed tomography revealed tumor emboli in the bilateral internal iliac veins and peritoneal dissemination around the liver and sigmoid colon. Docetaxel-gemcitabine chemotherapy (docetaxel at 70 mg/m^2^ on day 1, gemcitabine at 800 mg/m^2^ on days 1 and 8) was initiated the day of this finding. However, the tumor emboli in the bilateral internal iliac veins extended to the IVC, and the front edge was located close to the RA after one course of docetaxel-gemcitabine chemotherapy. We determined that the chemotherapy was not effective. Thus, pirarubicin-ifosfamide chemotherapy was initiated (pirarubicin at 40 mg/m^2^ on day 1, ifosfamide at 1.5 mg/m^2^ on days 1–4). Six cycles of this chemotherapy led to reductions in both the tumor emboli and peritoneal dissemination. The patient retained a good quality of life without any pain or dyspnea for 10 months after the initial surgery. However, the peritoneal dissemination in the pelvis gradually worsened despite treatment with pazopanib. The pelvic mass increased to a diameter of 5 cm, and tumor-induced low abdominal pain appeared 12 months after the initial surgery. The patient died of postrenal uremia due to the pelvic tumor 14 months after the initial surgery.

## 3. Discussion

Uterine leiomyosarcoma is a rare mesenchymal neoplasm estimated to occur in 0.30 to 0.67 per 100,000 women. MLMS of the uterus is an extremely rare variant of uterine leiomyosarcoma, and only 56 cases were reported from 1982 to 2013. Uterine MLMS reportedly occurs in a wide age range (20–86 years), and common symptoms include atypical genital bleeding, a pelvic mass, and abdominal pain. Grossly, MLMS has a gelatinous appearance and circumscribed border. Microscopically, the tumor contains a large amount of myxomatous stroma and invades adjacent tissues and/or the lumens of surrounding veins. Traditionally, the mitotic count has been used to distinguish leiomyosarcomas from leiomyomas. However, the diagnosis of MLMS is currently based on the gross and microscopic features of the tumor rather than the mitotic index [1].

Although intravenous leiomyomatosis is a well-known entity, the occurrence of intravenous extension of uterine leiomyosarcoma is extremely rare. To the best of our knowledge, only three other cases of intravenous uterine leiomyosarcomatosis have been described, and this is the first reported case of uterine MLMS [10–12]. In this case, the intravenous MLMS extended into the RA. Because intracardiac tumor emboli can cause dyspnea, heart failure, and sudden death, prompt and adequate surgical intervention is mandatory. However, the patient's clinical condition should be carefully considered because in such cases, radical operations involving the use of heart-lung machines are required for tumor resection.

No treatment of uterine MLMS has yet been established. Complete surgical excision appears to be an effective initial treatment. Burch and Tavassoli [2] reported that 1 of the 12 patients in their series underwent surgery for two recurrences and was disease-free at the last follow-up, 113 months after her initial diagnosis [2]. Similar cases involving slow tumor growth, surgical excision for repeated recurrences, and relatively long survival times have also been described [3, 4]. Although only described in a small number of patients, adjuvant radiation therapy and chemotherapy do not seem to be effective [1, 4–9]. Early Gynecologic Oncology Group studies, which included both leiomyosarcomas and carcinosarcomas, focused on either doxorubicin or doxorubicin with ifosfamide. Although one such study showed a tendency toward a reduced risk of recurrence, there was no survival benefit [13]. A subsequent Gynecologic Oncology Group study showed that fixed-dose gemcitabine plus docetaxel is a reasonable option for first-line treatment of uterine leiomyosarcoma and exhibited an objective response rate of 35.8% [13]. In our case, we selected pirarubicin-ifosfamide chemotherapy, which appeared to be effective. Pirarubicin is a derivative of doxorubicin and has less cardiotoxicity.

In conclusion, we have described a rare case of MLMS of the uterus with gross intravascular extension that reached the RA. Although complete surgical resection of the tumor is desirable, tumor reduction surgery followed by adjuvant chemotherapy might help to maintain a good quality of life.
